# Transformable Gaussian Reward Function for Socially Aware Navigation Using Deep Reinforcement Learning

**DOI:** 10.3390/s24144540

**Published:** 2024-07-13

**Authors:** Jinyeob Kim, Sumin Kang, Sungwoo Yang, Beomjoon Kim, Jargalbaatar Yura, Donghan Kim

**Affiliations:** 1 Department of Artificial Intelligence, College of Software, Kyung Hee University, Yongin 17104, Republic of Korea; wls2074@khu.ac.kr (J.K.); 1222kbj@khu.ac.kr (B.K.); 2 Department of Electronic Engineering (AgeTech-Service Convergence Major), College of Electronics & Information, Kyung Hee University, Yongin 17104, Republic of Korea; suminsk@khu.ac.kr (S.K.); p1112007@khu.ac.kr (S.Y.)

**Keywords:** Artificial Intelligence, machine learning, reinforcement learning, robotic programming, robots, reward shaping

## Abstract

Robot navigation has transitioned from avoiding static obstacles to adopting socially aware navigation strategies for coexisting with humans. Consequently, socially aware navigation in dynamic, human-centric environments has gained prominence in the field of robotics. One of the methods for socially aware navigation, the reinforcement learning technique, has fostered its advancement. However, defining appropriate reward functions, particularly in congested environments, holds a significant challenge. These reward functions, crucial for guiding robot actions, necessitate intricate human-crafted design due to their complex nature and inability to be set automatically. The multitude of manually designed reward functions contains issues such as hyperparameter redundancy, imbalance, and inadequate representation of unique object characteristics. To address these challenges, we introduce a transformable Gaussian reward function (TGRF). The TGRF possesses two main features. First, it reduces the burden of tuning by utilizing a small number of hyperparameters that function independently. Second, it enables the application of various reward functions through its transformability. Consequently, it exhibits high performance and accelerated learning rates within the deep reinforcement learning (DRL) framework. We also validated the performance of TGRF through simulations and experiments.

## 1. Introduction

Over the years, persistent interest has been shown in robot navigation within the field of robotics. Initially, research focused on basic obstacle avoidance and random navigation strategies [1,2,3]. Advances in navigation techniques have led to simultaneous localization and mapping (SLAM) [4,5,6], wherein robots estimate their positions and create maps for effective movement. Strategies have expanded to address dynamic environments [7,8,9,10,11,12,13,14,15,16,17], as robotics has consistently pursued advancements in navigation.

However, despite the coexistence of robots and humans, the effective avoidance of dynamic obstacles by robots remains a significant challenge. Unlike static environments [18], socially aware navigation requires the integration of perception, intelligence, and behavior, including adherence to social norms [19,20], necessitating the ability to differentiate between static objects and humans.

Therefore, two main research directions to address this challenge have emerged: reactive navigation [9,10,11,15] and navigation utilizing reinforcement learning (RL) [21,22,23,24]. Reactive navigation responds to real-time sensor data, with limitations in predicting future movements. RL employs the Markov decision process (MDP) and deep reinforcement learning (DRL) [12,13,14,16,17,25] to leverage deep neural networks for well-informed decisions and enable robots to navigate safely in human environments.

**Figure 1:**
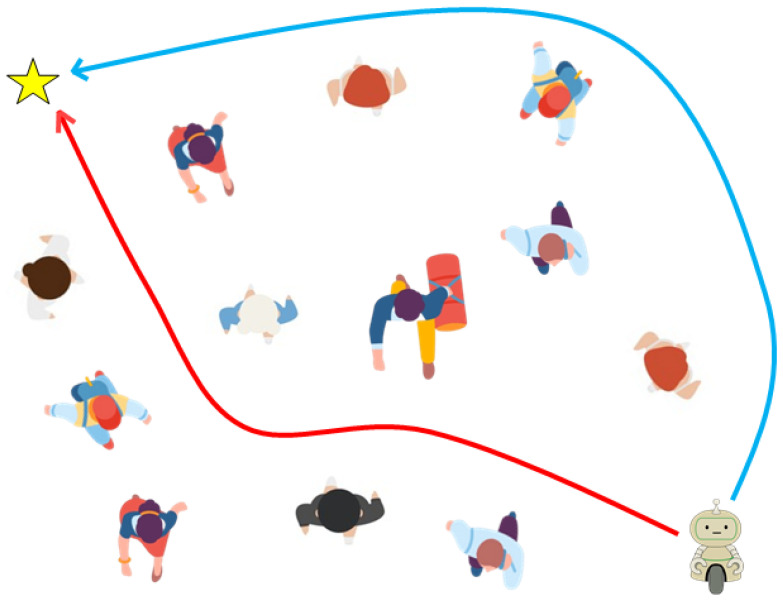
When the robot is equipped with inadequate reward functions in a crowd environment, it chooses a dangerous route (a red arrow). However, when a transformable Gaussian reward function (TGRF) is applied, the robot opts for a safe route (a blue arrow). The yellow star represents the goal.

However, the challenges in defining these reward functions become particularly evident in crowded environments [21,26,27,28]. These reward functions essentially serve as the guiding principles for steering the actions of agents by evaluating the potential value of each action. As a result, human-crafted rewards have become indispensable because they cannot be set automatically. However, as demonstrated in Figure 1, inadequately designed reward functions can induce risky behaviors in human-robot interactions. (The scenario in Figure 1 was simulated as described in Section 4). Moreover, the manual design of numerous rewards presents several critical issues.

First, the proliferation of distinct reward functions necessitates a redundant number of hyperparameters [12,13,14,16,17,25,29,30,31]. Each reward function requires tailored functions that align with its specific attributes, such as distance from humans, direction toward the goal, or even human intentions. This can lead to extensive fine-tuning of hyperparameters and reward imbalance issues. These problems can inadvertently steer robots toward humans, thereby increasing the risk of collisions [12]. Therefore, simplifying the reward functions and designing them for easy comparison and analysis is essential for enhancing human safety and improving robot performance.

Second, the fixed form of the reward function is neither temporally nor experimentally efficient. Static, context-specific reward functions have been used [12,14,16,17,32,33,34,35]; however, they often fail to adequately represent their unique characteristics. Even when the same formula is employed, diverse attributes may not be captured accurately. Addressing this discrepancy requires extensive empirical design and numerous experiments to achieve higher performance.

Third, the hurdles can be extended to effective learning [27]. Crafting appropriate reward functions remains a significant challenge, which may lead to collisions and hinder robot learning.

This paper proposes a transformable Gaussian reward function (TGRF) to address these issues. This approach makes several crucial contributions. (1) A smaller number of hyperparameters significantly alleviates the burden of parameter tuning. Each hyperparameter has a distinct role, making tuning and the search for the optimal reward function more efficient. (2) The TGRF demonstrated adaptability to various reward functions through dynamic shape adjustments. In this paper, by adjusting only one hyperparameter, various forms of reward functions can be created. Such adaptability is in stark contrast to previous models [13]. (3) The TGRF exhibits accelerated learning rates, notably in crowded environments, effectively harnessing the potential of DRL.

To demonstrate the performance of the TGRF, we introduce the key points in reward shaping and relevant papers for comparison in Section 2. In Section 3, we present background knowledge and characteristics of the TGRF and introduce the reward functions using the TGRF. In Section 4, we present two experiments conducted to demonstrate the performance of the TGRF and present the results of this study’s application to real environments; finally, we conclude the paper in Section 5.

## 2. Related Works

### 2.1. Integration of Prior Knowledge through Human-Delivered Reward Functions

RL is a machine learning approach operating within the MDP [21], where an agent interacts with a specific environment and receives rewards. The primary objective is to achieve the maximum cumulative reward. Therefore, the reward function significantly influences the agent’s decision-making process (policy).

However, in vast state spaces, the transitions between states and rewards may be unknown or stochastic because agents typically lack complete information about all aspects of the environment [36]. Moreover, the agent remains unaware of the consequences and outcomes of the actions until they are executed.

Consequently, in such scenarios, agents require substantial experience to converge on optimal policies for complex tasks in the absence of prior knowledge. To address this challenge, research on RL has explored reward shaping, aiming to guide agents toward making better decisions at appropriate times using suitable reward values [27,28]. This approach aims to significantly reduce the learning time by fostering convergence to optimal policies without explicit prior knowledge.

Previous studies extensively explored the incorporation of prior knowledge into reward functions [26,28,37,38,39,40]. However, crafting reward functions encompassing general prior knowledge, such as the apprehension of collision risks based on proximity to humans or progress relative to the destination, is challenging because of various environmental and psychological factors. These factors render it impossible to express knowledge simply through mathematical formulations.

To address this challenge, recent research has focused on utilizing inverse reinforcement learning (IRL), wherein humans intervene at the intermediate stages to provide rewards [41]. In addition, a study utilizing natural language to communicate intermediate reward functions with agents has emerged [42]. These studies involved humans evaluating the actions of robots as rewards. They demonstrated the transmission of rewards imbued with prior knowledge to agents during learning, thereby accelerating the learning process and enhancing algorithm performance.

However, because of their reliance on human intervention, these approaches are unsuitable for environments that require extensive learning or complex tasks without direct human involvement. Therefore, there is a growing need for research on reward shaping that considers prior knowledge and delivers high performance without direct human intervention.

### 2.2. Reward Function Analysis for Human Avoidance in Robot Navigation

In recent studies, reward functions commonly employ different formulas based on objectives without direct human intervention and can be broadly classified into four types [12,13,14,16,17,25,29,30,31]. These are rewards categorized as follows: reaching the destination, $r_{goal}\left(s_{t}\right)$; avoidance of collision with humans, $r_{col}\left(s_{t}\right)$; distance from humans, $r_{disc}\left(s_{t}\right)$; and distance from the destination, $r_{pot}\left(s_{t}\right)$.

$r_{goal}\left(s_{t}\right)$ and $r_{col}\left(s_{t}\right)$ typically assume consistent values. On the other hand, $r_{dist}\left(s_{t}\right)$ consistently imposes a larger negative reward as the distance between humans and robots diminishes using linear, L2 norm, or exponential functions. This design aligns with the psychological theory of proxemics [20], which evaluates discomfort based on interpersonal distances and integrates prior knowledge about the potential discomfort associated with varying distances between humans and robots.

In addition, $r_{pot}\left(s_{t}\right)$ incentivizes the robot’s faster arrival at the destination by applying positive/negative rewards based on changes in the L2 norm distance between the robot and the destination. These approaches reflect rational strategies by integrating prior knowledge of discomfort levels associated with distances (proxemics) and apprehension regarding the probability of collision with humans.

However, studies related to reward shaping and RL argue that it is crucial to verify whether rewards take appropriate forms and maintain suitable proportions [27,28]. If the shapes of the rewards are inadequate for the objectives or overly biased, the robot may steer its learning process in a direction not determined by the algorithm, potentially leading to the freezing robot problem [43]. In addition, an excessive number of hyperparameters may hinder the search for optimal performance.

The aforementioned studies experimentally determined the reward functions and counts of the hyperparameters. As a result, some were excessively simplistic, preventing researchers from intuitively adjusting rewards through hyperparameters, whereas others exhibited complex structures that hindered the straightforward modification of hyperparameters. This resulted in significant time consumption to achieve optimal performance and limitations in adjusting inadequate rewards, necessitating a redesign of the reward function.

For instance, in [13], researchers designed simple reward functions with redundant hyperparameters. This resulted in a substantial nine-fold difference between $r_{pred}\left(s_{t}\right)$ and $r_{disc}\left(s_{t}\right)$. This leads to situations in which the robot favors actions with smaller negative rewards from $r_{disc}\left(s_{t}\right)$ over larger negative rewards from $r_{pred}\left(s_{t}\right)$, thereby resulting in intrusion and collision.

This directly affects the learning process, rendering the task of identifying the appropriate reward function and hyperparameters more challenging and requiring formula modification.

However, the TGRF allows for intuitive and versatile applications with fewer hyperparameters. Enabling researchers to adjust rewards intuitively reduces the time required to explore suitable reward functions, and optimal performance can be ensured by finely tuning reward balances. To substantiate this claim, we directly compared the reward function used in socially aware navigation (SCAN) [16], a decentralized structural recurrent neural network (RNN) (DS-RNN) [17], the Gumbel social transformer + human–human attention (GST + HH Attn) [13,44,45,46], and crowd-aware memory-based RL (CAM-RL) [31].

## 3. Suggested Reward Function

In Section 3.1, we briefly introduce background knowledge regarding the model. In Section 3.2, we elaborate on the proposed TGRF. Finally, in Section 3.3, we describe the application of the TGRF to the reward functions within the environment and model of [13].

### 3.1. Markov Decision Process (MDP)

A Markov decision process (MDP) is a framework that mathematically represents information to solve problems using reinforcement learning. A MDP consists of six components: ⟨*S*,*A*,*P*,*R*,γ,$S_{0}$⟩. The state *S* represents information about the environment that influences the agent’s decisions. The action *A* refers to the behavior that the agent decides upon considering *S*. The transitional probability *P* represents the transition probability between the current state and the state at the next time step. In this study, this probability distribution is unknown to the agent. The reward *R* evaluates the agent’s action *A*. The discount factor gamma is used to adjust the value of future rewards. $S_{0}$ is the initial state.

In each episode, the individuals and the robot start from an initial position $s_{0}\in S_{0}$, and each selects actions $a_{t}\in A$ according to their respective policies $\pi (a_{t}|s_{t})$ at time step *t*. The robot then receives a reward $r_{t}\in R$ and transitions to the next state $s_{t+1}$ based on the transition probability $P(\cdot |s_{t},a_{t})$. If the robot collides with an individual, reaches its destination, or exceeds the maximum time *T*, the episode is terminated, leading to the beginning of a subsequent episode.

In this study, *S* includes the robot’s state information, denoted as $w^{t}$, and the positional information of humans. $w^{t}$ comprises the robot’s current position ($p_{x}$, $p_{y}$), velocity ($v_{x}$, $v_{y}$), destination ($g_{x}$, $g_{y}$), maximum velocity $v_{max}$, angle θ, and robot radius ρ. The positional information of humans includes current and future positions. Defining the positional information of the i−th person at time step *t* as $u_{i}^{t}$, $u_{i}^{t}$ consists of the person’s current position ($p_{x}^{i}$, $p_{y}^{i}$). Additionally, using a trajectory-prediction algorithm [44], we can predict future positions from time steps t+1 to t+K based on the positional information of humans. This predicted state information is defined as ${\hat{u}}_{1}^{t+1:t+K}$. Therefore, $s_{t}$ can be defined as $s^{t}=\left[w^{t},u_{1}^{t},{\hat{u}}_{1}^{t+1:t+K},\ldots ,u_{n}^{t},{\hat{u}}_{n}^{t+1:t+K}\right]$ encompassing $w^{t}$ and the positional information of humans from 1 to *n*.

Reward functions *R* can vary depending on the objective. Positive/negative rewards are given based on a design specified by the researcher at each time step. However, RL is not conducted based on individual reward values but rather on multiple accumulated rewards. Therefore, small changes in individual rewards influence the total sum of the rewards, the prioritization of the agent’s actions, performance, and learning speed. That is why meticulous reward shaping by the researcher is required, and parameter tuning is challenging for researchers. This paper proposes the TGRF as a powerful reward function to alleviate these problems.

### 3.2. Transformable Gaussian Reward Function (TGRF)

TGRF leverages the characteristics of a normal distribution [47], which allows it to transform into various shapes using only two hyperparameters, mean (μ) and variance (σ). This adaptability enhances the model’s flexibility to fit diverse prior knowledge and apply it to reward functions, reducing the burden on researchers for hyperparameter tuning and aiding in the swift identification of appropriate hyperparameters. The normal distribution, $N(\mu ,\sigma^{2})$, is symmetric around μ, peaks at μ, and its width is determined via σ, making it highly versatile in shape with just these two parameters.

(1) $$\begin{array}{c} TGRF\left(h_{TGRF},\sigma_{TGRF}\right)=\frac{h_{TGRF}\cdot N\left(\mu_{TGRF},\sigma_{TGRF}^{2}\right)}{C_{\mathrm{norm}}},\\ C_{\mathrm{norm}}=\max N\left(\mu_{TGRF},\sigma_{TRGF}^{2}\right) \end{array}$$

In (1), the TGRF involves three hyperparameters: $h_{TGRF}$, representing the weight of TGRF; $\mu_{TGRF}$, meaning the mean; and $\sigma_{TGRF}$, indicating the variance. However, in this work, we assumed that the mean $\mu_{TGRF}$ = 0 because $\mu_{TGRF}$ is less important than other hyperparameters and 0 in normal cases. Thus, the TGRF can actually be adjusted with two hyperparameters.

$C_{norm}$ ensures that the TGRF attains a maximum value of 1, irrespective of $\sigma_{TGRF}$. This allows the scaling of the TGRF solely by $h_{TGRF}$, enabling researchers to intuitively control its maximum value.

Note that the scaling of the reward function is closely related to the prioritization of actions mentioned in Section 3.1. The reward functions discussed in Section 2.2 were not normalized, and their scaling maximum values were adjusted with more than one hyperparameter. Consequently, it was challenging for researchers to experimentally balance these reward functions, leading to performance degradation, slower learning speeds, and the freezing robot problem [43]. We designed the TGRF to eliminate as many redundant hyperparameters as possible, allowing adjustments to be made independently with a single parameter, making it easier to adjust the balance.

**Figure 2:**
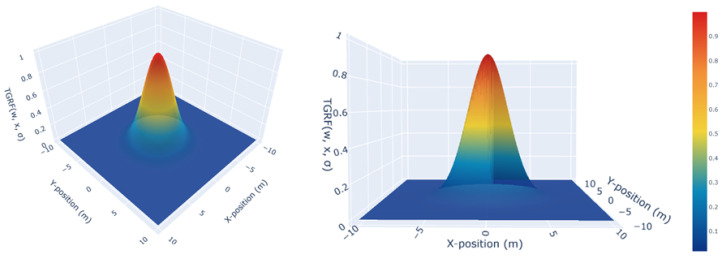
The TGRF. The X-axis denotes the X-position in meters, the Y-axis represents the Y-position in meters, and the Z-axis indicates negative reward when $h_{TGRF}=1$, $\sigma_{TGRF}=2$.

$\sigma_{TGRF}$ determines the transformability. As $\lim_{\sigma_{TGRF}\to \infty }TGRF\left(h_{TGRF},\sigma_{TGRF}\right)$, it takes on a constant form insensitive to changes in $x_{TGRF}$, while as $\lim_{\sigma_{TGRF}\to 0}TGRF\left(h_{TGRF},\sigma_{TGRF}\right)$, it resembles an impulse function. This versatility enables the creation of diverse forms of the TGRF, such as constant, linear, nonlinear, and Gaussian, that are adaptable to specific objectives. As a result, it ultimately represents a shape similar to that shown in Figure 2.

The reward functions described in Section 2.2 have limited flexibility. In particular, for reward functions that continuously change according to variables such as distance, finding the optimal shape is very challenging. Due to these problems, researchers must redesign and tune the reward functions to achieve better performance. The TGRF offers versatility in generating various shapes and better performance by only adjusting one hyperparameter. This significantly reduces the time and effort required by researchers while enabling fine-tuning to match the specific characteristics of objects.

**Figure 3:**
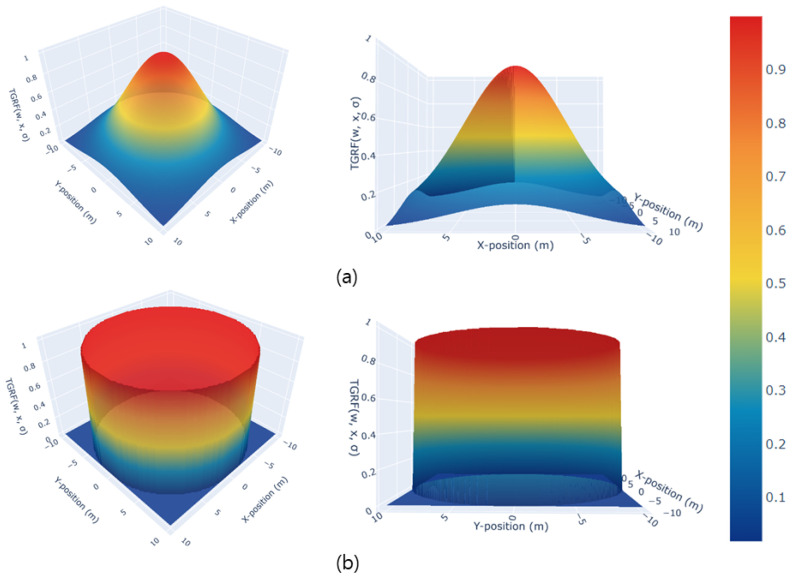
Transformability of the TGRF. The X-axis denotes the X-position in meters, the Y-axis represents the Y-position in meters, and the Z-axis indicates a negative reward. In (**a**), $h_{TGRF}=1$, $\sigma_{TGRF}=5$. In (**b**), $h_{TGRF}=1$, $\sigma_{TGRF}=5000$.

Figure 3 illustrates the creation of different shapes using the same TGRF by simply adjusting $\sigma_{TGRF}$. Figure 3a shows a TGRF that generates a continuous Gaussian distribution, making it suitable for moving objects or humans via the application of varying negative rewards. Figure 3b shows the discrete column-like shape. This configuration is suitable for stationary reward functions and objects. These reward functions will be demonstrated in practical applications in Section 3.3, and the results of their application will be shown in Section 4.2.

### 3.3. Application of Transformable Gaussian Reward Function (TGRF)

In this subsection, we present an example of applying the TGRF to the reward functions mentioned in Section 2.2.

Reward $r(s_{t},a_{t})$ is categorized into five types. First, $r_{goal}\left(s_{t}\right)$ = 10 represents the positive reward when the robot successfully reaches its destination. Second, $r_{col}\left(s_{t}\right)$ = −10 serves as a negative reward incurred upon colliding with another individual. Third, $r_{disc}\left(s_{t}\right)$ represents the negative reward for entering a danger zone. Fourth, $r_{pot}\left(s_{t}\right)$ corresponds to the positive/negative reward contingent on the change in distance to the destination $S_{goal}$. Finally, $r_{pred}\left(s_{t}\right)$ denotes the negative reward invoked when entering a prediction.

**Figure 4:**
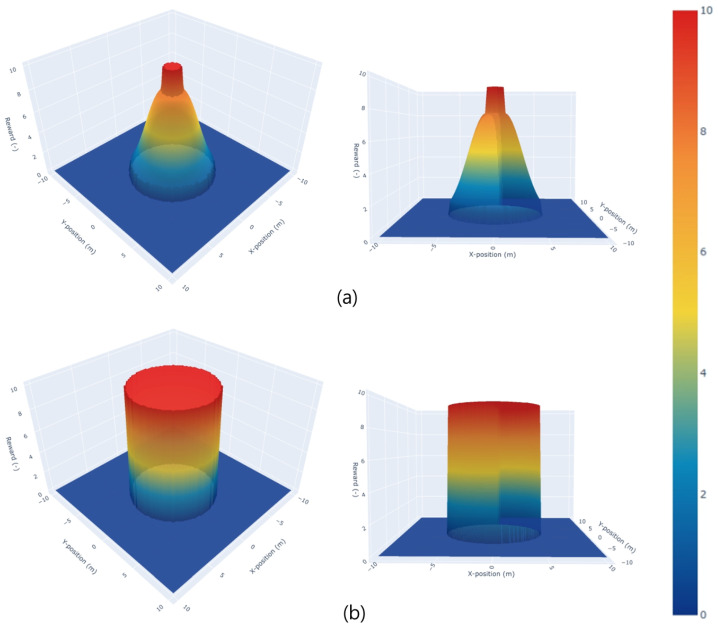
TGRF is applied to reward functions. The X-axis denotes the X-position in meters, the Y-axis represents the Y-position in meters, and the Z-axis indicates a negative reward value. The central cylinder represents $r_{col}$, and the surrounding distribution represents $r_{disc}\left(s_{t}\right)$. Beyond $d_{disc}$, $r_{disc}\left(s_{t}\right)$ becomes 0 ($s_{t}\notin S_{\mathrm{danger}\;\mathrm{zone}}$). In (**a**), $h_{TGRF}=8$, $\sigma_{TGRF}=3$. In (**b**), $h_{TGRF}=10$, $\sigma_{TGRF}=1000$.

$r_{disc}\left(s_{t}\right)$ is designed to prevent collisions with humans and maintain a safe distance. In Figure 4a, the negative reward $r_{disc}\left(s_{t}\right)$ is imposed when the robot enters the danger zone ($d_{min}$ is within $d_{disc}$) determined using the nearest human distance, denoted as $d_{min}$. The formula used is as follows:

(2) $$\begin{array}{c} r_{\mathrm{disc}}\left(s_{t}\right)=\mathrm{TGRF}\left(h_{\mathrm{disc}},\sigma_{\mathrm{disc}}\right) \end{array}$$

In (2), $h_{disc}$ and $\sigma_{disc}$ have different roles. Tuning $h_{disc}$ directly scales the reward function, establishing a linear correlation with $r_{disc}\left(s_{t}\right)$, thereby enabling adjustment of the overall reward balance to prioritize driving tasks. $\sigma_{disc}$ regulates the breadth of the Gaussian negative reward concerning the distance between humans and the robot. This enables the robot to react more sensitively or less sensitively to the distance from humans. Through experiments, we found that $h_{disc}$ is related to the probability of collisions with humans, while $\sigma_{disc}$ affects the understanding of human movements and intentions. Therefore, by tuning the hyperparameters according to the test cases, we were able to achieve higher performance than the baseline. (This will be shown in Section 4.2).

The potential reward $r_{pot}\left(s_{t}\right)$ represents the positive reward associated with the potential field and is defined as follows:

(3) $$\begin{array}{c} r_{\mathrm{pot}}\left(s_{t}\right)=TGRF\left(1.5\cdot \Delta d,\sigma_{\mathrm{pot}}\right),\\ \Delta d=(-d_{\mathrm{goal}}^{t}+d_{\mathrm{goal}}^{t-1}) \end{array}$$

$r_{pot}\left(s_{t}\right)$ plays a crucial role in guiding a robot toward its destination. However, high values of $r_{pot}\left(s_{t}\right)$ can lead to the positions of humans being ignored, while low values can lead to the freezing robot problem [21]. In addition, we found that continuously changing reward functions resulted in both of these drawbacks. Therefore, we aimed to maintain a constant TGRF as shown in Figure 4b by applying $h_{pot}$ = 1.5 ·Δd and high $\sigma_{pot}$.

$r_{pred}\left(s_{t}\right)$ is the negative reward for the prediction and is defined as follows:

(4) $$\begin{array}{c} r_{\mathrm{pred}}^{i}\left(s_{t}\right)=\min_{k=1,\ldots ,K}\left(1_{i}^{t+k}\frac{r_{\mathrm{col}}}{2^{k}}\right),\\ r_{\mathrm{pred}}\left(s_{t}\right)=\min_{i=1,\ldots ,n}r_{\mathrm{pred}}^{i}\left(s_{t}\right) \end{array}$$

In (4), $r_{pred}\left(s_{t}\right)$ is used only in models that employ trajectory predictions. $r_{pred}\left(s_{t}\right)$ denotes the negative reward value when the robot is positioned along the trajectory of the *i*-th person. $1_{i}^{t+k}$ indicates whether the robot is in the predicted position of the *i*-th person at time t+k or not. Thus, $r_{pred}\left(s_{t}\right)$ takes the smallest negative reward among all individual trajectory negative rewards that the robot takes. In our experiments, we adopted $r_{pred}\left(s_{t}\right)$ from [13] to demonstrate the performance enhancement even with different reward functions.

The final definition of the reward function is as follows:

(5) $$r\left(s_{t},a_{t}\right)=\left\{\begin{array}{cc} +10, & \mathrm{if}\;s_{t}\in S_{\mathrm{goal}}\\ -10, & \mathrm{if}\;s_{t}\in S_{\mathrm{collision}}\\ r_{\mathrm{pred}}\left(s_{t}\right)+r_{\mathrm{disc}}\left(s_{t}\right), & \mathrm{if}\;s_{t}\in S_{\mathrm{danger}\;\mathrm{zone}}\\ r_{\mathrm{pred}}\left(s_{t}\right)+r_{\mathrm{pot}}\left(s_{t}\right), & \mathrm{otherwise} \end{array}\right.$$

In summary, the TGRF offers the distinct advantage of intuitive and efficient modification of the reward function with fewer hyperparameters. This enables the robot to make rational decisions and reduces time-consuming fine-tuning tasks.

## 4. Simulation Experiments

### 4.1. Environment and Navigation Methods

#### 4.1.1. Simulation Environment

We employed a 2D environmental simulator as in previous studies [13]. This simulator features a 12 × 12 m space with a 360° field of view and 5 m sensor range for LiDAR. A fixed number of humans (20) was used to represent a crowded setting.

(6) $$\begin{array}{cc} p_{x}\left[t+1\right] & =p_{x}\left[t\right]+v_{x}\left[t\right]\Delta t,\\ p_{y}\left[t+1\right] & =p_{y}\left[t\right]+v_{y}\left[t\right]\Delta t \end{array}$$

Both humans and robots were operated using holonomic kinematics to determine their velocities $\left(a_{t}=\left[v_{x},v_{y}\right]m/s\right)$ along the x- and y-axes. Holonomic kinematics refers to a state in which degrees of freedom can move independently without any constraints. This implies that robots and machines can move without limitations on their position or orientation. As a result, the action space of a robot is continuous, allowing both robots and humans to immediately achieve their desired speed within a time frame of Δt, assuming they operate within the maximum speed limit. Therefore, the positions of humans and robots are continuously updated, according to (6).

The robot has attributes such as a size of ρ=0.3 m and a maximum speed of $v_{max}$ = 1.0 m/s. Humans also have characteristics such as a size ranging from 0.3 to 0.5 m and a maximum speed varying between 0.5 and 1.5 m/s. In addition, the locations and destinations of the robot and humans were randomized, and the destinations were set to not be excessively close. Humans perform subsequent actions based on their own characteristics and information about the current positions and velocities of others.

#### 4.1.2. Navigation Methods

To demonstrate the superiority of the TGRF across various models, experiments were conducted using a total of five learning-based models employed in [13,17]:

- DS-RNN: A model utilizing an RNN. However, it does not predict trajectories.
- No pred + HH Attn: Attention-based model excluding trajectory prediction ($r_{pred}=0$).
- Const vel + HH Attn: The experimental case assumes that the trajectory-prediction algorithm predicts the trajectories to move at a constant velocity.
- Truth + HH Attn: This experiment assumes that the robot predicts the actual human trajectory.
- GST + HH Attn: Scenarios in which the robot predicts the human trajectory nonlinearly using the GST.

However, in the simulations, humans moved using a reaction-based model, which is different from the aforementioned methods. Humans exchanged their location information with each other and calculated velocities based on their positions, engaging in collision avoidance by altering their speed and direction using ORCA and SF [9,10,11].

Random seeding was applied during training, resulting in varying outcomes for each training episode. To handle the varying outcomes, multiple training runs were conducted with a total time step of 2 × $10^{7}$ for the DS-RNN [17] and 1 × $10^{7}$ for the other algorithms [13,16,31]. The learning rate was set to 4 × $10^{-5}$ for all policies. Subsequently, test data were acquired from 500 test episodes. The evaluation metrics applied to the test data included the success rate (SR), average navigation time (NT) in seconds, path length (PL) in meters for successful episodes, and intrusion time ratio (ITR).

The complete source code is available at https://github.com/JinnnK/TGRF, accessed on 18 February 2024.

### 4.2. Results

#### 4.2.1. Results in Different Navigation Methods

**Figure 5:**
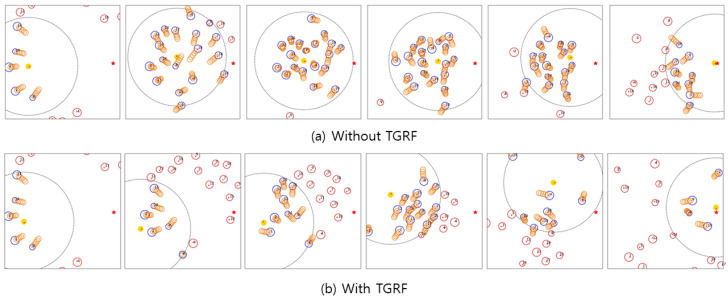
Comparison of scenarios with and without the TGRF. Yellow circles represent robots, blue circles represent humans within the sensor range, red circles represent humans outside the sensor range, and orange circles in front of the blue circles indicate trajectories predicted by the GST.

We compared the performance of the TGRF to the performance of the reward function presented in [13]. Table 1 shows the performance when individuals adhered to ORCA, whereas Table 2 outlines the performance when adhering to SF. The hyperparameters were set to $h_{disc}=0.25$, $\sigma_{disc}=0.2$, $d_{disc}=0.5$, $h_{pot}=1.5$, and $\sigma_{pot}=1000$. From Table 1 and Table 2, and Figure 5, we can identify three impacts of the TGRF.

First, the TGRF results in higher performance by adjusting only two hyperparameters. In previous research [13], there was a significant deviation between each reward value. This led to the agent ignoring actions with low reward values, resulting in the agent failing to learn appropriate actions for different situations and thus producing a low SR.

In contrast, TGRF maintained an appropriate balance between each reward by adjusting $h_{TGRF}$, guiding the agent to choose actions suitable for the situation. As a result, SR improved in most navigation methods. Notably, Table 1 shows that the TGRF achieved an average SR of 94.091%, whereas [13] attained only 77.1% with the GST + HH Attn policy, marking a notable 17% increase.

On the other hand, DS-RNN [17] showed poor performance regardless of the reward. This is analyzed to be due to the navigation method lacking sufficient information required for learning, given the complexity of the environment, preventing convergence to an optimal policy.

Second, TGRF demonstrates suitable performance for socially-aware navigation. ITR represents the proportion of time during which the robot invades a safe distance from people throughout the entire navigation. In Table 1, TGRF showed improved ITR performance in all metrics. This improvement is attributed to TGRF’s formula based on proxemics [20] and a Gaussian distribution, unlike the linear $r_{disc}$ in [13].

This signifies that the TGRF effectively incorporates prior knowledge based on the role of the reward function, indicating resilience in the freezing robot problem [43]. Consequently, it is evident that the robot demonstrates high performance by taking appropriate actions according to the situation.

Third, the TGRF leads to enhanced recognition of human intent and collision avoidance. Figure 5a shows the robot’s route when TGRF was not applied. The robot struggled when confronted with crowds. Notably, in the test cases, the robot ventured into the crowd, resulting in unintended collisions with humans while attempting to navigate the crowd. This means that the robot selected aggressive or impolite behaviors, such as sidestepping, to avoid human and unintentional collisions or made risky decisions to reach a destination faster, resulting in collisions. This behavior reflects a deficiency in understanding the broader intentions of humans.

However, the robot in Figure 5b proactively positioned itself behind the crowd before converging at a single point. This means that reward functions using TGRF were well balanced, enabling the robot to navigate effectively without colliding with individuals.

Notably, when Truth + HH Attn was used, ref. [13] showed an SR of 5% as the policy did not converge optimally, whereas TGRF showed a high learning speed with an SR of 92% under the same amount of training episodes.

Consequently, this section signifies that the TGRF effectively incorporates prior knowledge and that its priorities are well integrated into the policy. This suggests that the performance of the algorithm can be further enhanced when the TGRF is applied. Further evidence of this enhancement is reflected in the results in Table 1 and Table 2, where the average SR and standard deviation show similar or superior performance compared with previous iterations. In the other test cases, we observed that the robot selected a more secure and effective route rather than a faster and more dangerous route.

#### 4.2.2. Performance Comparison with Other Reward Functions

In this section, we applied the reward functions from other studies [13,16,31] introduced in Section 2.2. The navigation method employs GST + HH Attn. Participants adhered to the ORCA approach by recording SR, NT, PL, and ITR every 2000 episodes. The total number of episodes conducted was 20,000 for the GST + HH Attn. In this section, two characteristics of TGRF can be identified.

**Figure 6:**
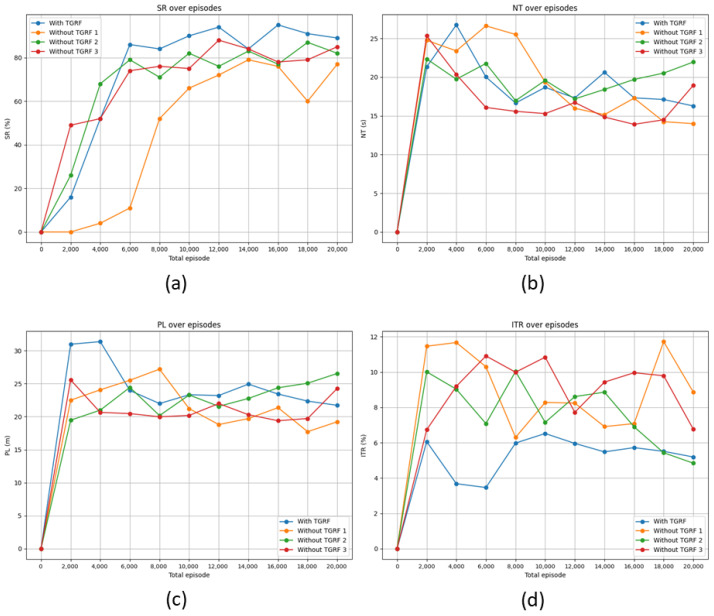
Comparison of performance among four types of reward functions. The blue line represents the reward function incorporating the TGRF, while the orange line corresponds to the reward function in [13], green reflects [16], and red signifies [31]; (**a**) denotes SR, (**b**) represents NT, (**c**) stands for PL, and (**d**) signifies ITR.

First, we observed that the TGRF led to an overall performance improvement compared with the other reward functions. As shown in Figure 6a, the TGRF was able to drive the algorithm’s performance up to a maximum of 95% over 16,000 total episodes. Conversely, the other reward functions achieved a maximum SR of 90%. This indicates that the TGRF harmonizes appropriately with the other reward functions, assuming a shape that aligns with the role of the rewards, thereby eliciting the algorithm’s maximum performance.

As depicted in Figure 6b, the NT of TGRF decreased with repeated learning, ultimately confirming the second lowest NT. Correspondingly, in Figure 6c, the second lowest PL was observed. This is associated with the ITR, as lower NT and PL imply that the robot tolerates negative rewards owing to $r_{disc}\left(s_{t}\right)$ reaching the destination, resulting in a higher ITR. For instance, in the orange graph, the highest ITR, along with the lowest NT and PL values, can be observed. As shown in Figure 5a, this leads to the choice of shorter and riskier paths, increasing the likelihood of not understanding human intentions and a higher possibility of collisions. However, as shown in Figure 6d, the model incorporating the TGRF maintained the lowest ITR in most cases. This demonstrates that the TGRF selects the most efficient and safe paths compared to the other models while maintaining the highest SR, reflecting the intentions of the algorithm, as shown in the results of Figure 5b.

Second, Figure 6 demonstrates the significant advantage of the TGRF in terms of learning speed compared to the other reward function. As shown in Figure 6a, the three models reached saturation after 6000 episodes. At this point, the model with the TGRF achieved the highest SR. This indicates that the TGRF contributes to faster learning speeds.

However, the TGRF has limitations in crowded environments. It does not inherently enhance the performance of the core algorithms. Comparable performance was achieved for certain policies, as shown in Table 1 and Table 2. This suggests that the TGRF expedites the algorithm to achieve optimal performance rather than enhancing the algorithm itself.

## 5. Real-World Experiments

This study extended beyond simulations to real-world experiments. The model trained using the unicycle approach was applied to a physical robot in a real environment. Unicycle kinematics presents limitations in terms of direction and position control when compared to holonomic kinematics. Our experimental setup consisted of a host computer equipped with an Intel (Santa Clara, CA, USA) i5-10600 processor at 3.30 GHz and an NVIDIA (Santa Clara, CA, USA) RTX 3070 GPU, which was integrated with a Turtle-bot3 (Seongnam-si, South Korea). A LiDAR sensor, LDS-01 (Seongnam-si, South Korea), played a pivotal role in human detection and robot position estimation. Robot positioning relies on SLAM localization, and human detection is accomplished using a 2D people detection algorithm based on 2D LiDAR data [35].

Although our study assumed the absence of static obstacles other than humans, our real-world experiments were conducted in a confined space measuring approximately 3 × 5 m with static obstacles. These experiments involved scenarios in which the robot navigated between predefined start and destination points and encountered one to four pedestrians along its path. The maximum speed was approximately 0.6 m/s, and the investigation covered scenarios involving four moving individuals.

**Figure 7:**
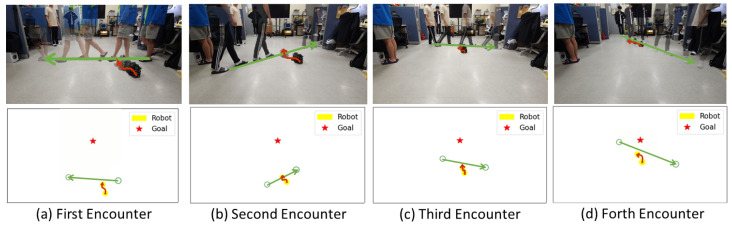
Evasive actions performed by a robot in real-world scenarios and corresponding renderings with four humans (from left to right). Green arrows represent the movement path of humans, while red arrows indicate the movement path of the robot. In addition, yellow circles indicate robots, green circles represent humans, and red stars indicate destinations. These illustrations showcase avoidance strategies employed by the robot as it encounters successive individuals: (**a**) first, (**b**) second, (**c**) third, and (**d**) fourth human.

As shown in Figure 7a, the robot faces diagonally upward as the pedestrian moves from right to left. In this scenario, the robot rotated to the left, aligning with the pedestrian’s direction of movement, instead of moving behind (to the right) the human. This decision appears rational because both the destination and the robot’s current orientation are oriented diagonally upward, making a leftward maneuver the most efficient choice when considering the human direction, speed, and destination. Notably, in another experiment involving three individuals, the robot was observed to halt temporarily instead of moving to the left.

In Figure 7b, the robot encounters a pedestrian walking diagonally from left to right. In response, the robot navigates to the left to avoid obstructing the path of the pedestrian.

In Figure 7c, the robot faces a human walking from left to right. Similarly, it predicts the human’s trajectory and executes a leftward turn to avoid collisions while approaching the destination.

In Figure 7d, the robot encounters a human crossing diagonally from left to right near the destination. The robot smartly avoids humans by initially turning left, avoiding the pedestrian, and then turning right to reach its destination.

These actions underscore the robot’s ability to make real-time decisions based on a dynamic environment, considering factors such as the human path, velocity, and proximity to the destination. The robot’s avoidance strategies prioritize efficiency while maintaining safety and are influenced by various factors, including its current orientation and the overall context of the situation. These real-world experiments verified the adaptability of the model in complex and dynamic environments, where human-robot interactions necessitate responsive and context-aware behavior. Comprehensive renderings and additional experimental videos are available at https://youtu.be/9x24k75Zj5k?si=OtczdVXPUnbGwpv-, accessed on 30 August 2023.

Two primary limitations were encountered during this experiment. The first is the computation load: The use of DNNs for action and trajectory predictions significantly increases the computational demands. Considering the number of pedestrians, particularly for trajectory prediction, the time required for the next action was approximately 0.22 s. This resulted in irregular robot movements and delayed pedestrian responses. The second are the physical constraints: The accuracy of human detection and prediction is affected by sensor noise, limitations in detection performance, and challenges in determining human angles. These factors lead to occasional misidentification of obstacles as humans or limitations in the precision of human location information, thereby reducing the accuracy of trajectory prediction. In addition, noise from the LiDAR sensor and location information errors caused by the movement of the robot accumulated over time, resulting in inaccuracies in the location values as the experiment progressed.

## 6. Results and Future Research

This paper introduces a TGRF specifically designed for robots navigating crowded environments. The TGRF offers several advantages, including high performance with minimal hyperparameters, adaptability to diverse objectives, and expedited learning and stabilization processes. These claims are supported by the success rates achieved and the algorithm’s enhanced ability to discern human intentions when compared to previous reward functions.

However, challenges have emerged in both the simulations and real-world experiments. In the simulations, these challenges involved sensitivity to hyperparameters, algorithmic limitations, a trade-off correlation between SR and NT, and the absence of static obstacles. In the real-world tests, the challenges included sensor noise and physical constraints.

Hence, in future research, we propose two key strategies. First, we will apply the TGRF to various environments and different objects. While our study demonstrates its effectiveness primarily with human rewards, we plan to expand our experiments by applying the TGRF to diverse objects, such as walls, obstacles, and drones. Second, we will devise a TGRF that considers physical limitations. Although the TGRF performs exceptionally well under ideal conditions, its performance decreases in reality due to computational load and physical issues. Therefore, we aim to implement a dynamically adaptive TGRF that adjusts according to the situation by incorporating knowledge regarding these physical limitations.

## Figures and Tables

**Table 1:** Navigation results using the reward function from [13] and the TGRF. Humans followed ORCA.

Reward	Navigation Method	Mean (Sigma) of SR	SR (%)	NT (s)	PL (m)	ITR (%)
	DS-RNN	35.5 (7.697)	44.0	20.48	20.58	15.45
	No pred + HH Attn	59.636 (4.848)	67.0	17.49	20.30	17.22
Without	Const vel + HH Attn	65 (8.023)	81.0	17.34	21.95	6.15
TGRF	Truth + HH Attn	5.545 (1.616)	5.0	21.60	23.89	14.83
	GST + HH Attn	77.1 (5.718)	88.0	14.18	20.19	7.38
	DS-RNN	30.5 (4.843)	40.0	27.11	25.19	12.19
With	No pred + HH Attn	59.364 (6.692)	72.0	18.17	21.92	14.16
TGRF	Const vel + HH Attn	87.909 (3.029)	92.0	16.38	22.33	5.08
(Ours)	Truth + HH Attn	84.909 (4.776)	92.0	17.13	22.52	5.30
	GST + HH Attn	94.091 (2.843)	97.0	17.63	23.81	3.92

**Table 2:** Navigation results using the reward function from [13] and the TGRF. Humans followed SF.

Reward	Navigation Method	Mean (Sigma) of SR	SR (%)	NT (s)	PL (m)	ITR (%)
	DS-RNN	29.8 (5.231)	36.0	23.26	27.13	13.38
Without	No pred + HH Attn	12.091 (8.062)	28.0	26.52	34.98	12.78
TGRF	Const vel + HH Attn	92.182 (3.588)	96.0	14.74	21.49	5.24
	GST + HH Attn	91.636 (2.267)	95.0	13.74	20.47	5.37
	DS-RNN	48.6 (9.013)	62.0	22.48	25.26	10.14
With	No pred + HH Attn	77.364 (6.079)	87.0	16.19	21.95	13.43
TGRF	Const vel + HH Attn	95.273 (1.911)	98.0	17.00	23.55	5.39
(Ours)	GST + HH Attn	92.909 (3.579)	96.0	15.37	21.91	5.81

## Data Availability

The data presented in this study are openly available in Github at https://github.com/JinnnK/TGRF, release v1.0., accessed on 18 February 2024.
